# Non-pharmacological interventions for the management of perinatal anxiety in primary care: a meta-review of systematic reviews

**DOI:** 10.3399/BJGPO.2023.0022

**Published:** 2023-08-09

**Authors:** Victoria Silverwood, Laurna Bullock, Joanne Jordan, Katrina Turner, Carolyn A Chew-Graham, Tom Kingstone, Shoba Dawson

**Affiliations:** 1 School of Medicine, Keele University, Keele, UK; 2 Centre of Academic Primary Health Care, Bristol Medical School, University of Bristol, Bristol, UK; 3 Midlands Partnership NHS Foundation Trust,Trust Headquarters, St George’s Hospital, Stafford, UK; 4 Applied Research Collaboration (ARC) West Midlands, Keele University, Keele, UK

**Keywords:** perinatal anxiety, pregnancy, systematic review, meta-review, narrative synthesis, interventions, primary health care, general practitioners

## Abstract

**Background:**

Perinatal anxiety (PNA), anxiety that occurs during pregnancy and/or up to 12 months postpartum, is estimated to affect up to 21% of women, and may impact negatively on mothers, children, and their families. The National Institute for Health and Care Excellence (NICE) has called for further research around non-pharmacological interventions in primary care for PNA.

**Aim:**

To summarise the available international evidence on non-pharmacological interventions for women with PNA in a primary care population.

**Design & setting:**

A meta-review of systematic reviews (SRs) with narrative synthesis was performed following Preferred Reporting Items for Systematic Reviews and Meta-Analyses (PRISMA) guidance.

**Method:**

Systematic literature searches were conducted in 11 health-related databases up to June 2022. Titles, abstracts, and full-text articles were dual-screened against pre-defined eligibility criteria. A variety of study designs were included. Data were extracted about study participants, intervention design, and context. Quality appraisal was performed using the AMSTAR 2 tool (A MeaSurement Tool to Assess systematic Reviews). A patient and public involvement group informed and contributed towards this meta-review.

**Results:**

Twenty-four SRs were included in the meta-review. Interventions were grouped into the following six categories for analysis purposes: psychological therapies; mind–body activities; emotional support from healthcare professionals (HCPs); peer support; educational activities; and alternative or complementary therapies.

**Conclusion:**

In addition to pharmacological and psychological therapies, this meta-review has demonstrated that there are many more options available for women to choose from that might be effective to manage their PNA. Evidence gaps are present in several intervention categories. Primary care clinicians and commissioners should endeavour to provide patients with a choice of these management options, promoting individual choice and patient-centred care.

## How this fits in

PNA is anxiety that occurs during pregnancy or up to 12 months postpartum. Current NICE guidance recommends that women with PNA are offered a choice of pharmacological therapy, psychological therapies, or a combination of both, and has called for further research into non-pharmacological interventions for PNA. This meta-review has demonstrated that there are many more options that could be discussed with women that might be effective to help manage their PNA. Primary care clinicians and commissioners should endeavour to provide patients with a choice of these management options, promoting individual choice and patient-centred care.

## Introduction

PNA is defined as anxiety that occurs during pregnancy and/or up to 12 months after delivery.^1^ Global prevalence of PNA is estimated to be as high as 21%,^2^ higher than perinatal depression (PND), which is estimated to affect 11.9% of perinatal women.^3^ PNA may occur as a single condition or be comorbid with other perinatal mental health (PMH) disorders such as PND.^4^ Despite its high estimated prevalence, PNA may be underdiagnosed and therefore often undertreated.^5^

Evidence around the potential adverse consequences of PNA is conflicting;^6^ however, PNA has been linked to adverse outcomes for pregnancies^7–9^ and ongoing risks for mothers,^1,9,10^ children,^11–13^ and surrounding family.^14,15^ Currently, the leading cause of perinatal mortality is death by suicide, which can be preceded by PNA as well as other PMH conditions.^16^ PNA may also have negative consequences for wider society owing to financial costs linked to increased need to access public services and loss of productivity.^17^

The 2016 *Five Year Forward View for Mental Health* ^18^ outlined greater investment in PMH services to improve access to interventions for women with PMH problems. The *NHS Long Term Plan* ^19^ built on this, establishing PMH referral pathways and increasing community and inpatient services. While some women may experience severe PNA symptoms and require inpatient or secondary care treatment, the majority of women with PNA are supported by primary care or by community PMH services.^1^

NICE clinical guidance (CG192) for antenatal and postnatal mental health has outlined recommendations for treatment of people with PNA with pharmacological therapies, psychological therapies, or a combination of both.^1^ Recent meta-analyses have suggested there is insufficient evidence to confirm that antidepressants cause harm to the developing foetus or breastfeeding child;^20,21^ however, women have reported decisional conflict around choosing to take medication to manage their PNA symptoms and have expressed preference for non-pharmacological options.^22,23^ Therefore, NICE has called for further research into non-pharmacological interventions for PNA.

Alongside psychological therapies, a growing number of non-pharmacological interventions are described in the literature that could offer valid options for PNA management in primary care. Previously, there has been insufficient evidence around these interventions to determine their clinical effectiveness, so they are not currently reflected in clinical guidance and are therefore not discussed with women as management options for PNA.

This meta-review synthesises evidence from existing SRs of non-pharmacological interventions for PNA to address the following three key aims: (1) demonstrate the range of potential available non-pharmacological interventions for women with PNA in a primary care population; (2) summarise the available international evidence on different interventions, including whether there is currently sufficient evidence to determine their clinical effectiveness; and (3) understand which interventions might be acceptable to women with PNA.

## Method

A meta-review is a type of SR that comprehensively synthesises evidence from multiple SRs to answer a specific research question, often relating to clinical interventions.^24^ This meta-review was conducted and reported following the PRISMA guidelines.^25^

### Patient and public involvement and engagement

VS and TK met virtually with a PMH patient and public involvement and engagement (PPIE) group (*n* = 4 experts by experience) twice. Initially, the PPIE group reflected on the different interventions that women may choose to access, referring to their personal experiences, peer reviewed literature, and relevant grey literature before contributing to the development of the research question and the protocol design. Following data synthesis, VS presented the results and the PPIE team discussed whether the interventions outlined were consistent with their experiences of supporting women with PNA. PPIE members received payment for their time.

### Search strategies

Search strategies were developed and tested with support from an information and SR expert (JJ). Twelve healthcare-related databases were searched via Ovid and EBSCOhost from 2000 to June 2022 (see supplementary material for databases and sample search strategy). A combination of MeSH headings and free-text terms relating to the perinatal period, PNA, and different intervention types were used. VS hand-screened reference lists of the included SRs and performed a citation search, including reviews and key articles by leading PMH researchers.

### Screening process

Database search results were imported into RefWorks reference management software and duplicates removed. VS screened all titles and abstracts, and LB screened a 20% sample, referring to a pre-defined eligibility criteria (see Table 1) for inclusion. There was high inter-rater reliability score (kappa coefficient ≥0.80) between reviewers. Both reviewers independently reviewed the full text of the remaining articles and SRs where at least 50% of included primary studies specifically focused on PNA were included. Discrepancies were resolved through discussion between the reviewers and the wider team if necessary. Translation was sought for four articles not published in English.

**Table 1.** Eligibility criteria following Population Intervention Comparison Outcome (PICO) format

**Population or participants and conditions of interest**	Perinatal women aged ≥18 years with anxiety (either self-identified or HCP-identified) or anxiety and depression
**Interventions**	Any systematic review that reviews an intervention aiming to reduce, treat, or manage anxiety during the perinatal period, which could be: medical (not pharmacological) psychological social other A variety of study designs are of interest, so systematic reviews that report the following study designs will be included: RCTs controlled clinical trials cohort studies case-control studies qualitative studies
**Comparisons or control groups**	Any control group, which could be intervention versus usual or standard care in the perinatal period.
**Outcomes of interest**	Symptoms of anxiety during the perinatal period, which can be self-reported or measured using standardised anxiety assessment tool such as Generalised Anxiety Disorder-7 (GAD-7) or Stait-Trait Anxiety Inventory (STAI).Patient experiences and/or perspectives of being treated for PNA.*NB: Many articles report anxiety alongside depression; in these instances, data specifically focusing on PNA have been extracted. If data are presented in combination, for example, 'anxiety with depression' then these have not been extracted*.
**Setting**	Studies based in primary or community care.
**Study designs**	Any systematic review that reviews primary qualitative, quantitative, or mixed-methods studies.At least 50% of the studies reported within the systematic review must have anxiety-specific outcomes.
**Exclusion criteria**	systematic reviews of studies outside of the perinatal period systematic reviews of studies that review interventions for perinatal mental health conditions other than anxiety or anxiety with depression (for example, studies that exclusively describe interventions for conditions such as PTSD and OCD and not for comorbid anxiety narrative reviews that are not systematic in nature (for example, do not follow the PRISMA guidelines) reviews that report case studies and/or case series systematic reviews that review studies that evaluate pharmacological interventions

HCP = healthcare professional. OCD = obsessive compulsive disorder. PRISMA = Preferred Reporting Items for Systematic Reviews and Meta-Analyses. PTSD = post-traumatic stress disorder. RCT = randomised controlled trial.

### Data extraction and quality assessment

Data at review level were extracted independently by both VS and LB then compared. Included SRs were quality assessed independently by two reviewers (VS [100%], LB [50%], and SD [50%]) using the AMSTAR 2 tool,^26^ which assesses the methodological quality of SRs (see Table 2.) Any discrepancies were resolved through discussion.

**Table 2.** Assessment of methodological quality of the included systematic reviews using the AMSTAR 2 tool. *(A non-colour dependent version of this table is available to download from the supplementary materials)*

**Domain**	1	2	3	4	5	6	7	8	9	10	11	12	13	14	15	16	
**Review**																	**Confidence in reviews**
Ashford *et al* 2016^30^											N/A	N/A			N/A		Moderate
Bayrampour *et al* 2019^31^																	Critically low
Callanan *et al* 2022^32^											N/A	N/A			N/A		High
Desai *et al* 2021^33^																	Moderate
Dhillon *et al* 2017^34^																	High
Domínguez-Solís *et al* 2021^35^											N/A	N/A			N/A		High
Evans *et al* 2018^36^																	Moderate
Evans *et al* 2020^53^											N/A	N/A					High
Evans *et al* 2022^37^																	Moderate
Hall *et al* 2016^38^											N/A	N/A					High
Hall *et al* 2020^39^																	High
HTA report 2021^40^																	Moderate
Lau *et al* 2017^41^																	Moderate
Lau *et al* 2021^42^																	High
Lever-Taylor *et al* 2016^43^																	Low
Loughnan *et al* 2019^44^																	High
Maguire *et al* 2018^45^																	Low
Marc *et al* 2011^50^											N/A	N/A					High
Matvienko-Sikar *et al* 2016^46^											N/A	N/A					Low
Matvienko-Sikar *et al* 2021^47^											N/A	N/A			N/A		Low
Mueller and Grunwald 2021^51^											N/A	N/A			N/A		Low
Sánchez-Polán *et al* 2021^52^																	Low
Shi *et al* 2017^48^											N/A	N/A					Critically low
Yan *et al* 2022^49^																	Moderate

Domains: 1. research questions and inclusion criteria include the components of PICO? 2. explicit statement that the review methods were established prior to the conduct of the review and justify significant deviations from the protocol 3. explain their selection of the study designs for inclusion 4. use of a comprehensive literature search strategy 5. study selection in duplicate 6. data extraction in duplicate 7. provided list of excluded studies and justification 8. included studies described in adequate detail? 9. satisfactory technique for assessing the risk of bias (RoB) 10. sources of funding for the studies 11. If meta-analysis was performed: appropriate methods for statistical combination of results 12. If meta-analysis was performed: assess the potential impact of RoB in individual studies 13. account for RoB in individual studies when interpreting/ discussing the results of the review? 14. explanation for, and discussion of, any heterogeneity observed 15. If they performed quantitative synthesis: publication bias (small study bias) and discuss its impact 16. report any potential sources of conflict of interest, including funding received

Code for AMSTAR 2 tool: Critical domain = BLUE. Yes = GREEN. Partial yes = YELLOW. No = RED. Not applicable = WHITE.

### Data synthesis

Significant heterogeneity between the included SRs regarding study designs, intervention types, and outcome measures was anticipated; a meta-analysis was therefore not appropriate, and a narrative synthesis was conducted^27^ and reported following Synthesis Without Meta-analysis (SWiM) guidance.^28^

## Results

### Study characteristics

**Figure 1.**
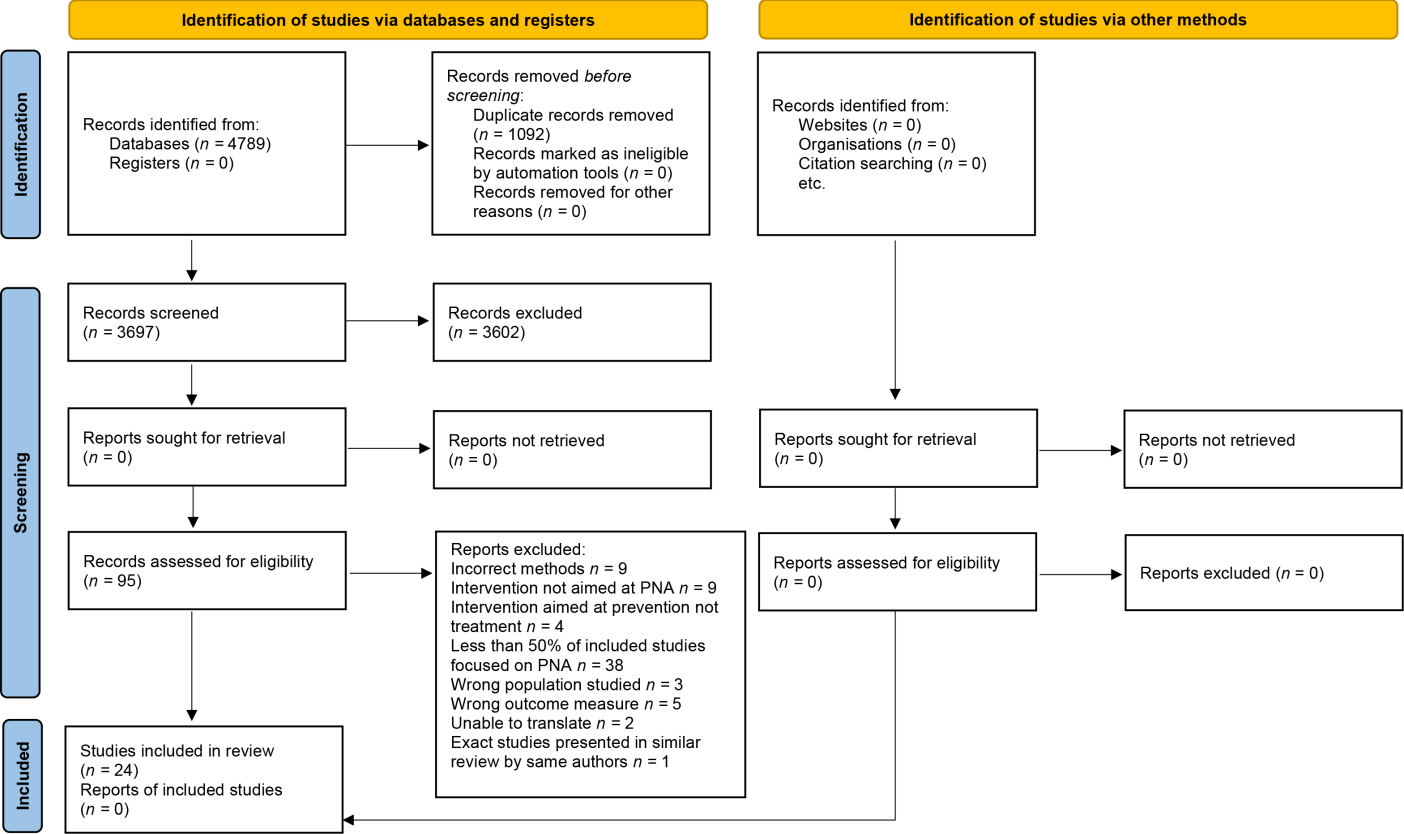
PRISMA flowchart. PNA = perinatal anxiety

Database searches identified 4789 records. After removing duplicates, 3697 titles and abstracts were screened. Ninety-five full texts were read, and a total of 24 SRs included. Figure 1 shows the flowchart.^29^

This meta-review provides an international perspective as SRs included data from the UK, US, Canada, Australia, New Zealand, Germany, Switzerland, Belgium, The Netherlands, Greece, Portugal, Sweden, Poland, Hong Kong, Korea, China, Iran, India, and Taiwan. Twenty-three SRs^30–52^ presented quantitative data; two of these 23 presented both quantitative and qualitative data;^37,43^ and one SR presented only qualitative data.^53^ Participant numbers within SRs ranged between 146 and 5156. Supplementary Table S1 provides an overview of the SR characteristics.

Quality appraisal of included SRs conducted using AMSTAR2 ^26^ ranged from ‘critically low’ to ‘high’. SRs were mainly rated as critically low or low because they did not explicitly report on the development of a study protocol or discuss how or if they addressed publication bias. However, these domains are unlikely to affect the results presented in the SRs and contributed to the available evidence on non-pharmacological interventions for PNA.

### Types of intervention

Some SRs focused on a specific type of intervention to manage PNA, such as psychological therapies, whereas others were interested in a variety of non-pharmacological management options for PNA. To allow for comparison, the interventions discussed in the included SRs were grouped into six intervention categories following consideration of their clinical application and mirroring categories presented in two included SRs.^36,53^ Supplementary Table S2 provides an overview of the intervention type included within each SR and Supplementary Table S3 outlines the results. A brief summary of results is presented in Table 3.

**Table 3.** Summary of quantitative results

Interventioncategory	Type of interventions within category	Number of SRs presenting data for this intervention category	Synthesis of SR author conclusions
Psychological therapies	CBT MBIs psychodynamic therapy ACT BA IPT	18^1–18^	10 SRs concluded that psychological therapies could be effective at treating PNA: CBT,^9,10,14^ MBIs,^3,12,15,17^ and CBT and/or MBIs.^2,5,11^8 SRs discussed inconsistent evidence,^1,4,6–13,16,18,18^ with two SRs calling specifically for further research into psychological therapies for PNA.^1,13^
Mind–body activities	active relaxation guided imagery biofeedback heart-rate biofeedback hypnotherapy imagery joint mobility exercises meditation muscle strengthening Pilates prayer relaxation therapy tai chi yoga	7^5,6,12,15,16,19,20^	5 SRs presented data in favour of use of mind–body activities for PNA.^5,6,16,19,20^Two SRs gave no narrative summary for extraction.^12,15^
Emotional supportfrom HCPs	home visits from HCPs telephone support and home visit from HVs	2^5,6^	One SR concluded could be beneficial.^6^One SR discussed lack of sufficient data to develop conclusion.^5^
Peer support	telephone-based peer support	1^6^	SR concluded there was insufficient data to determine if it could be considered effective.
Educational activities	antenatal education mindfulness childbirth and parenting programme motivational interviewing diet or exercise education psychoeducation remote antenatal education self-guided book reading transition to parenthood education programme	7^3,5–9,12,15,15^	3 SRs presented data in support of antenatal education to manage PNA^3,5,9^ with one of those querying if this would be clinically relevant.^9^4 SRs did not provide a conclusion as to the benefit of educational activities.^6,7,12,16^
Alternative or complementary therapies	acupressure acupuncture essential oils massage music therapy probiotic supplement capsules	5^6,7,12,16,21^	3 SRs suggested massage therapy could be effective for treating PNA.^5,22,23^1 SR suggested that probiotic therapy could be helpful but also called for further research to confirm this.^21^1 SR concluded that both acupuncture and acupressure could be effective across the perinatal period for PNA.^6^1 SR presented data and suggested that essential oils, aromatherapy, and music therapy could be beneficial for managing PNA.^5^

ACT = acceptance and commitment therapy. BA = behavioural activation. CBT = cognitive behavioural therapy. HCPs = healthcare professionals. HVs = health visitors. IPT = interpersonal psychotherapy. MBIs = mindfulness-based interventions. PNA = perinatal anxiety. SR = systematic reviews

### Psychological therapies

Within the meta-review, 18 SRs presented data around psychological therapies for PNA.^30–34–38–38,40–49^ Therapies discussed included cognitive behavioural therapy (CBT), interpersonal psychotherapy (IPT), mindfulness-based interventions (MBIs), behavioural activation (BA), psychodynamic therapy, and acceptance and commitment therapy (ACT), and were delivered face-to-face or remotely via electronic-health methods.

The majority of SRs presented evidence in support of the use of psychological therapies such as CBT,^40,41,45^ MBIs,^32,43,46,48^ and CBT and/or MBIs.^31,35,42^ The remaining SRs presented narrative summaries that were inconclusive around psychological therapies.^30,34,36–44,47,49,49^ Two SRs specifically called for further primary studies to be conducted,^30,44^ which contrasts with current clinical guidance recommendations.^1^

### Mind–body activities

Seven SRs discussed mind–body activities for PNA.^35,36,43,46,47,50,52^ These included physical activity (PA) during pregnancy such as yoga, tai chi, Pilates, hypnotherapy, imagery, meditation, and biofeedback.

A Cochrane review concluded that mind–body activities might be useful for both preventing and treating antenatal anxiety,^50^ and specific interventions that were reported to be effective in different SRs included PA during pregnancy,^52^ heart-rate biofeedback,^47^ and yoga.^35^ Delivery of mind–body activities appeared to be more effective when delivered by trained instructors rather than self-guided.^36^ Two SRs did not provide any specific narrative synthesis for extraction.^43,46^ Overall, the evidence presented to support the use of mind–body activities for PNA was positive.

### Emotional support from healthcare professionals

Two SRs discussed the impact of emotional support from HCPs for managing women with PNA.^35,36^ One suggested that home visits from HCPs, such as nurses and health visitors, to carry out activities, such as supportive listening, could be beneficial.^36^ The other SR presented data from one primary study, so did not present any conclusions.^35^ This meta-review did not find any additional evidence of any other SRs that discussed HCP support specifically for PNA, so there is a clearly identified evidence gap around this intervention in addition to usual care from HCPs.

### Peer support

Only one of the included SRs presented discussion around the impact of peer support on management of PNA. Data were reported from one primary study that concluded peer support was beneficial from their results but, as there were no further studies to review the SR, the authors highlighted that further research was required before conclusions could be reached.^36^ As with HCP support, further research is needed around peer support specifically for PNA.

### Educational activities

Seven SRs discussed the impact of face-to-face and electronically delivered educational activities for managing PNA.^32,35–40,43,46,46^ Three SRs provided narrative summary discussions, which concluded that antenatal education in particular seemed to be effective for managing PNA;^32,35,40^ however, one questioned if their results were clinically relevant.^40^ For the remaining four SRs, despite mentioning educational programmes, there were limited or no data to extract.^36,37,43,47^ Overall, the perspective of the SRs is that educational activities may be of benefit for helping to manage PNA.

### Alternative or complementary therapies

Five SRs discussed alternative or complementary therapies for PNA.^33,35,36,39,51^ Three SRs suggested that massage therapy was an effective option.^35,39,51^ One SR focused on the effectiveness of probiotic supplementation and suggested this could be a treatment option for PNA while calling for further RCTs to explore this therapy.^33^ One SR suggested that acupuncture and acupressure is effective across the perinatal period,^36^ and another reported small effect sizes for the use of both essential oils, aromatherapy, and music therapy.^35^

Although not routinely utilised or recommended in the UK, there is a body of evidence that suggests in the right context, various alternative or complementary therapies could be an option to support PNA management.^33,35,36,39,51^

### Acceptability of non-pharmacological interventions for perinatal anxiety

Three SRs within the meta-review reported qualitative data.^37,43,53^ Evans *et al* presented a qualitative SR that explored women’s views on the acceptability and effectiveness of various remote interventions for PNA. They presented data around the following four main themes: motivation and barriers to participation in studies; acceptability of interventions; satisfaction with interventions; and the perceived benefit of interventions.^53^ They reported that women’s views around the acceptability of different intervention types were generally positive; a finding that is consistent among all three of the SRs reporting qualitative data in this meta-review.^37,43,53^

Data presented highlighted that women valued having the opportunity to choose between therapies delivered in a group setting or individually,^43,53^ and it was important for women to feel safe, supported, and welcomed if they did choose an intervention that was delivered in a group setting.^53^ Two SRs acknowledged that there was benefit for women who were supported by trained professionals to learn more about PNA, how to accept their current life circumstances, and how to manage their emotions and mental wellbeing.^43,53^ One SR discussed data around women’s perceptions of the acceptability of suggested interventions and highlighted that the requirement for participation needed to avoid being ‘onerous’ and needed to fit into women’s lives.^37^

Overall, qualitative evidence suggested that women perceived a range of interventions could be effective and were acceptable when they were presented with choice, and when interventions could be adapted to suit individual life circumstances and context.

### Patient and public involvement and engagement perspectives

The PMH PPIE group reviewed the findings of the review and agreed that a more comprehensive range of options for PNA should be available; acknowledging individualised experiences of women with PNA. The lack of evidence included within the review around interventions offered by the voluntary sector and the limited evidence around the positive impact of peer support was discussed. This contrasts with the grey literature that promotes PMH peer support^54^ and the PPIE groups’ opinion that in their experiences, women regularly seek peer support for PNA.

## Discussion

### Summary

This meta-review has provided a summary of the available international evidence on non-pharmacological interventions for women with PNA in a primary care population. It has also provided primary care clinicians with a greater range of interventions they could discuss with women with PNA.

### Strengths and limitations

This meta-review has provided a global perspective on non-pharmacological options for PNA in primary care populations. A comprehensive, systematic search strategy was developed with an experienced information specialist and the searches were not limited to English-only articles. Two reviewers performed screening and data extraction with high inter-rate reliability scores. The meta-review has reported mixed-methods evidence, including quantitative and qualitative SRs.

The SRs in this meta-review included a wide variety of interventions, populations, and outcomes, so a meta-analysis was not conducted, and a narrative synthesis was used to combine results from the included SRs. There were some methodological challenges with regards to data extraction. Some SRs did not present relevant data for extraction, and data in several SRs could not be extracted as they included studies not relevant to this meta-review (for example, outcomes relating to tokophobia rather than PNA). Despite seeking translations for articles not written in English, it was not possible to have two articles translated.^55,56^ The overall quality of SRs included was critically low to high according to AMSTAR2^26^ and limited the reliability of some of the results of the SRs.

There was some overlap of individual studies included in multiple SRs; currently there is no standardised method to address this issue in meta-reviews.^57^ Overlap has the potential to introduce bias in meta-analyses where data from individual studies are double-counted.^58^ In this meta-review, the aim was not to estimate a pooled effect size but to explore which interventions and their elements might benefit women with PNA, and therefore study overlap has less impact.

### Comparison with existing literature

This international meta-review demonstrated that a variety of interventions, in addition to pharmacological and psychological therapies, have been evaluated for PNA and could potentially be utilised in UK primary care to manage PNA. Evidence around the use of psychological therapies is well established and the findings of this meta-review are consistent with existing literature.^1^ This review has also suggested that mind–body activities and alternative or complementary therapies could be effective, but that evidence gaps still exist for emotional support from HCPs, peer support, and educational activities.

### Implications for research and practice

Currently, NICE clinical guideline CG192 recommends pharmacological and/or psychological therapies to manage PNA.^1^ This meta-review has demonstrated that more options should be made available for women to choose from, as these might be effective and acceptable interventions to support management of their PNA.

In primary care, as well as offering psychological therapies, clinicians could discuss mind–body activities, and alternative or complementary therapies as options. Additional research focusing on emotional support from HCPs, peer support, and educational activities is needed before they could be formally recommended in guidance. However, clinicians could explore these options with women as they each appear to hold potential to help manage PNA.

Women may want to choose to access more than one intervention type and may express a preference for in-person care, electronic-health care, or a combination of both. There is currently a tension between what might be helpful to women and what is commissioned, and this should be addressed in future policy decisions around PNA interventions.

Qualitative data presented in this meta-review has highlighted that women value being able to choose from a range of intervention options to decide which suit their individual lives. It is important for clinicians to consider patients’ personal and social circumstances in order to offer person-centred care. It is important to consider how primary care clinicians can support women to access interventions that might be helpful to the individual women, but which are not yet commissioned in their localities. Further stakeholder perspectives around women’s preferences for different intervention types should be considered when commissioning decisions are made by NHS integrated care boards and primary care networks.

There is a wide range of potential interventions that could be offered to women to help them manage PNA. Primary care clinicians should be aware of these intervention options in order to provide patients with choice and promote individualised, person-centred care.

## Trial registration number

A protocol was developed and registered on PROSPERO: CRD42021202611.

## Data

All the data relied on for the conclusions of this research are available in the article and its supplementary materials.
